# Waiting for attention and care: birthing accounts of women in rural Tanzania who developed obstetric fistula as an outcome of labour

**DOI:** 10.1186/1471-2393-11-75

**Published:** 2011-10-21

**Authors:** Lilian T Mselle, Thecla W Kohi, Abu Mvungi, Bjørg Evjen-Olsen, Karen Marie Moland

**Affiliations:** 1 School of Nursing, Muhimbili University of Health and Allied Sciences, Dar es Salaam, Tanzania; 2 Department of Sociology and Anthropology, University of Dar es Salaam, Dar es Salaam, Tanzania; 3 Centre for International Health, Bergen, Norway and Department of Obstetrics and Gynaecology, Sørlandet Hospital, Kristiansand, Norway; 4 School of Nursing, Bergen University College, Bergen, Norway

## Abstract

**Background:**

Obstetric fistula is a physically and socially disabling obstetric complication that affects about 3,000 women in Tanzania every year. The fistula, an opening that forms between the vagina and the bladder and/or the rectum, is most frequently caused by unattended prolonged labour, often associated with delays in seeking and receiving appropriate and adequate birth care. Using the availability, accessibility, acceptability and quality of care (AAAQ) concept and the three delays model, this article provides empirical knowledge on birth care experiences of women who developed fistula after prolonged labour.

**Methods:**

We used a mixed methods approach to explore the birthing experiences of women affected by fistula and the barriers to access adequate care during labour and delivery. Sixteen women were interviewed for the qualitative study and 151 women were included in the quantitative survey. All women were interviewed at the Comprehensive Community Based Rehabilitation Tanzania in Dar es Salaam and Bugando Medical Centre in Mwanza.

**Results:**

Women experienced delays both before and after arriving at a health facility. Decisions on where to seek care were most often taken by husbands and mothers-in-law (60%). Access to health facilities providing emergency obstetric care was inadequate and transport was a major obstacle. About 20% reported that they had walked or were carried to the health facility. More than 50% had reported to a health facility after two or more days of labour at home. After arrival at a health facility women experienced lack of supportive care, neglect, poor assessment of labour and lack of supervision. Their birth accounts suggest unskilled birth care and poor referral routines.

**Conclusions:**

This study reveals major gaps in access to and provision of emergency obstetric care. It illustrates how poor quality of care at health facilities contributes to delays that lead to severe birth injuries, highlighting the need to ensure women's rights to accessible, acceptable and adequate quality services during labour and delivery.

## Background

The leading cause of death and disability among women of reproductive age in low-income countries are complications of pregnancy and childbirth. Access to skilled birth care has been identified as a major condition to lower maternal morbidity and mortality.

### Maternal mortality

In 2008, an estimated 342,900 women globally died from causes related to pregnancy, childbirth or unsafe abortion [1]. This represents a significant decrease from the WHO estimate of 535,900 deaths in 2005 [2]. While encouraging, this decrease was not experienced in sub-Saharan Africa where trained health care staff assist less than 50% of women in labour [3] and maternal morbidity and mortality remains high with more than 640 maternal deaths reported per 100 000 live births in 2008 [4]. With the majority of deaths occurring in this region, sub-Saharan Africa still carries the greatest burden of maternal deaths [1].

Nearly all maternal deaths could be avoided if affordable and good-quality obstetric care were available on a continuous basis [5]. A few developing countries have managed to significantly reduce the number of maternal deaths [6,7]. In Tanzania, the prevalence of maternal mortality has decreased from 610 per 100,000 live births in 1990 to 449 per 100,000 in 2008. Nonetheless, even with this reduction, the present estimated level is still unacceptably high [1]. Complications during labour, delays in seeking and receiving care often lead to poor outcomes for both mother and child, including maternal deaths, stillbirths, neonatal illnesses, and long-term complications such as obstetric fistula [8,9]

### Health care distribution and utilisation

In Tanzania, as in other countries in sub-Saharan Africa, the distribution and utilisation of health care facilities are limited [10-12]. With an emphasis on equity, the national health policy in Tanzania aims to secure access to quality primary and reproductive health care for all [13]. Birth care should be offered free at all public and private health care facilities at all levels, from local dispensary, health centre, district hospital and regional to national referral hospitals [14]. The country has a basic health care infrastructure extending into peripheral rural areas. Estimates indicate that about 80% of the population has access to health services, and that over 90% of the population live within 10 km of a health facility [14].

There is, however, an urban bias in the distribution of health facilities [3,15,16], negatively affecting availability of health care in general and emergency obstetric care (EmOC) for women residing in rural areas [17]. Furthermore, inadequate supplies [18] and serious human resources shortages [19] continue to compromise the quality of services offered. For example, each nurse and midwife serves nearly 4000 individuals, whereas each obstetrics and gynaecology specialist serves a population of about 400,000 [20]. The vast majority of skilled health personnel work in the large cities.

The key indicator for monitoring the country's progress in achieving the Millennium Development Goal (MDG) 5, to reduce maternal mortality by 75% by 2015 is the proportion of births attended by skilled personnel. In Tanzania, the utilization of health facilities for birth care is surprisingly low, compared to antenatal attendance, which is stable and high. While 94% of pregnant women attended antenatal clinics for check-ups in 2005 [3], only 43% gave birth in health facilities [21], and in 2006, only 36% of births were attended by a skilled attendant [22].

### Obstetric fistula

Obstetric fistula is a hole between a woman's birth passage and one or more of her internal organs, usually the bladder or the rectum. It may develop after obstructed labour, when the pressure of the baby's head against the mother's pelvis cuts off blood supply to delicate tissues until it causes necrosis. Obstetric fistula is one of the most severe childbirth injuries that occur when labour is allowed to progress for a period lasting from several days to a week without quick intervention, usually a caesarean section [23-26]. Women affected by obstetric fistula suffer from physical problems, but also from social ostracism and economic hardship [27-29]. Estimates indicate that more than 2 million women worldwide live with vesico vaginal fistula (VVF) or recto vaginal fistula (RVF) and the majority of these reside in Africa and Asia [24,30].

Recent data on obstetric fistula in Tanzania show that there are between 2,500 and 3,000 new cases each year [31], which is higher than earlier estimates of about 1,200 per year [32], indicating that the problem had been underestimated. However, because these estimates are institutionally based, it is likely that the increase is due to a rise in the number of affected women visiting health facilities for treatment.

Efforts to manage obstetric fistula in Tanzania began in the 1970s, but were formally institutionalised in 1996 when the Bugando Medical Centre (BMC) started a training programme for local surgeons on fistula surgery. By 2001, 50 hospitals reported conducting fistula surgery and some had special wards or units for obstetric fistula patients [32]. In addition, the African Medical and Research Foundation (AMREF) developed a fistula programme, which contributed substantially to the development of an effective and comprehensive strategy to address fistula in Tanzania. Many gynaecologists and nurses have been trained to handle fistula cases [33] and fistula surgery is now provided free of charge. Active case finding programmes, which trace patients in rural areas and bring them to hospitals for treatment, as well as an outreach programme to perform simple fistula repair, are in place [34].

### Study aims

The few studies carried out on obstetric fistula in Tanzania primarily assessed the availability of the obstetric fistula surgery services [32]. Further, some studies assessed population-based incidence and prevalence of obstetric fistulas [35], or focused on prevention, treatment and repair [36-39]. Only a few studies have been published on the dimensions of living with fistula and the associated social vulnerability of affected women [29,31], or have assessed urinary and reproductive health and quality of life following fistula surgery [40,41].

To our knowledge, a mixed methods study of the birth care experiences of women who developed VVF or RVF as a result of labour has not been conducted in Tanzania. The aim of this study was to use both qualitative and quantitative research methods to explore the birthing experiences of women affected by obstetric fistula, and barriers to accessing adequate quality of care during labour and delivery.

### Theoretical framework

Availability, accessibility, acceptability and quality of care (AAAQ) concept [42] and the three delays model [9] were used to understand the birth care experiences of women who developed obstetric fistula after prolonged labour. The AAAQ concept operationalises the right to health in terms of availability, accessibility, acceptability and quality of care. It asserts that to realise the right to health, AAAQ must be ensured at all levels of care. Adequate health infrastructure and services must be available within a geographical area, accessible physically and economically, acceptable culturally and ethically, and be of adequate medical quality. Failure to ensure the AAAQ of emergency obstetric care (EmOC) causes delays in accessing adequate health care that may be fatal [43]. Much as we understand the feasibility of the four-delay model described by the White Ribbon Alliance [44], the three delays model by Thaddeus and Maine [9] was used in this study because the data collected were well-fitted for it. The model identifies barriers that can delay access in the different phases of birth care that can prevent maternal mortality. These include delays in individual decision making to seek care, in identifying and getting to a health facility, and in obtaining adequate care after arriving at the facility. The three phases of delay are closely connected. The barriers and poor care encountered in reaching, and receiving care affect subsequent decision-making to seek care.

Drawing on both quantitative and qualitative data, the current study sought to explore women's experiences in the three phases of seeking birth care, specifically focusing on (1) decision making before arrival at a health facility, (2) barriers to reaching a health facility, and (3) barriers to access adequate care in a health facility. Exploration and identification of these issues will provide knowledge to the Ministry of Health and Social Welfare (MoHSW), Nurses' and Midwives' Council and other stakeholders, on formulation of policies and guidelines for the care of women and attainment of MDG 4 on child health and survival and MDG 5 on maternal health.

## Methods

### Study design

A mixed qualitative and quantitative method of concurrent dominant status design [QUAL+quan] was employed [45]. This method involves conducting a study with these two methodologies that occur approximately at the same time, such that one approach is given greater emphasis. In this study, the qualitative component is dominant. Data were collected simultaneously from different samples, analysis was done separately, and the combination occurred in the interpretation phase. Mixing qualitative and quantitative methods was important to enhance our understanding of the birth care experiences of women affected by obstetric fistula.

### Study setting

This study took place between October 2008 and February 2010 at the Comprehensive Community Based Rehabilitation (CCBRT) in Dar es Salaam and Bugando Medical Centre (BMC) in Mwanza. CCBRT and BMC hospitals were selected because they are the major service points for fistula surgery, and the researcher was ensured access to women affected by fistulas. The qualitative study was done at CCBRT, whereas the quantitative study was done at both CCBRT and BMC. CCBRT hospital is a private non-governmental organisation (NGO) and a major service delivery point for obstetric fistula repair located in Kinondoni district in Dar es Salaam. About 200 VVF and RVF operations are performed each year. The hospital has a fistula ward with a capacity of 21 beds, and a hostel where fistula patients live while awaiting fistula surgery. BMC is a consultant teaching hospital for the Lake and Western zones, situated in Mwanza city. As a referral centre for tertiary specialist care, it receives patients from six regions: Mwanza, Mara, Kagera, Shinyanga, Tabora and Kigoma. It has 900 beds and a special ward dedicated to fistula repair and recovery, with a capacity of 70 beds. On average, more than 300 women with obstetric fistula are treated annually.

### Informants and data collection

#### I). Qualitative study

Sixteen women affected by obstetric fistula who met the inclusion criteria, and provided written consent were recruited between October 2008 and April 2009. The inclusion criteria were women who were admitted to the hospital either before or after surgical fistula repair, who could speak Kiswahili, and who were willing to participate in the study. A senior nurse midwife identified women who met the inclusion criteria, explained the purpose and the method of the study including principles of confidentiality, and arranged for a suitable time for an interview. The first author (LTM) conducted all sixteen face-to-face interviews in Kiswahili, the national language of all informants. The interviews were conducted in the hospital out of view and hearing of patients and staff on the fistula ward.

An interview guide was prepared and revised during the course of data collection. It included topics and probing questions on women's social and demographic background, and on their experiences of seeking and receiving care during the process of labour and birth. Although many informants struggled with their feelings during the interview, almost all managed to tell their stories in an open and comprehensible manner. Interviews were conducted until data saturation had been reached in terms of no new information and themes obtained [46]. The length of the interviews, which were audio-taped, varied between 45 minutes and 2 hours.

#### II). Quantitative study

All women admitted in the fistula wards during the quantitative data collection period of July 2009 and February 2010, were asked to participate. One hundred fifty-one (151) women who provided informed consent were enrolled. Two research assistants (a senior nurse midwife and a nurse teacher) with experience in health research were trained to conduct the interviews; one in each hospital collected data using close-ended questionnaires (see additional File 1). The questionnaire was prepared by the research group for use in Ethiopia, Sudan and Tanzania, and was translated from English to Kiswahili. A pilot test of the questionnaire was done at Muhimbili National Hospital before the start of the main study.

### Data analysis

#### I). Qualitative study

Content analysis approach [47] was used to analyse the interviews. All interviews were transcribed and translated from Kiswahili to English by a linguistic teacher from Dar es Salaam University College of Education (DUCE); this made data accessible to non-Swahili speaking members of the research team. The translated scripts were read and compared with the original Swahili transcripts, and errors were corrected accordingly. Further, to ensure accuracy of translation, another person back-translated two interviews and found there were no significant differences.

The interviews were read several times to provide a sense of the material. This was followed by extraction of meaning units from transcripts. These were condensed by shortening the original text while maintaining the central meaning. The condensed versions were later assigned codes, which were grouped into categories depending on their similarities and differences. The interviewer always cross-referenced between Kiswahili and English transcriptions to ensure validity of the meaning units, codes and categories.

For this article, the focus was on delays, and thus all texts from interviews were analysed for identification of delay patterns of women's experiences of accessing obstetric care. The research team crosschecked the analysis, discussed and agreed on sorting of codes and naming of categories.

#### II). Quantitative study

Data were analysed using Statistical Package for the Social Sciences (SPSS) version 15 for Microsoft Windows. For descriptive analyses, frequencies and proportions were used to summarise the data. Cross-tabulation and chi-square statistics were used to assess the statistical significance of associations between variables. Quantitative data helped to enhance and complement findings from in-depth interviews [48].

### Ethical consideration

The Muhimbili University of Health and Allied Health Sciences (MUHAS) Research and Ethical Review Board approved the study. Permission to conduct interviews was obtained from CCBRT and BMC Hospitals. Written informed consent was obtained from all women affected by obstetric fistula in the qualitative study. The ethical committee approved the use of oral consent in the quantitative study since many of the women could neither read nor write.

Women were briefed about the objectives and procedures of the study. They were also informed about their right to agree or refuse to participate in the study and their right to withdraw from the study at any time. Special permission was obtained from informants in the qualitative study to use a tape-recorder during the interview. It was made clear that information provided, whether orally or in writing, would be treated with strict confidentiality and would only be used for the research purposes. All names used are fictitious.

## Results

After a brief presentation of the socio-demographic background of the study participants, a woman's experiences of seeking birth care in rural Tanzania introduced the problem of delays. Four categories emerged from the women's descriptions: deciding where to give birth, struggling to reach a health facility, waiting at the health facility, and receiving unskilled care (Table 1). Category 1 corresponds with the first delay, category 2 with the second delay, and categories 3 and 4 with the third delay.

**Table 1:** Codes and categories from content analysis of birthing experiences of women affected by obstetric fistula

Category	Deciding where to give birth	Struggling to reach a health facility	Waiting at health facility	Receiving unskilled care
**Codes**	-Had no money -Husband and mother in-laws decided to wait -Afraid of embarrassment -Parents told me to wait -Birth care sought from TBAs	-Facilities too far -No transport -No fare for transport -Walked for many hours -Bad roads-rainy season	-Assisted by relatives -Cared by TBAs -Waited for many hours -No doctor or medical assistant to give referral letter -Felt alone -Fear of death -No ambulance	-Blocked mouth -Exerting pressure on the abdomen -Tightened legs -Slapped -Forced to push -Felt severe pain -Referred in critical condition

Barriers to access health care were further elaborated through quotes from the qualitative interviews and quantitative results from the survey, focusing on decision making at home, transport to a health facility and care after arrival. The findings reveal a series of delays in the process of seeking and accessing adequate birth care.

### Characteristics of the Informants

The 16 women who were interviewed about their birth care experiences described themselves as peasants (n = 13), petty businesspersons (n = 2) or homemakers (n = 1). They were between 18 and 43 years of age, seven were either divorced or separated, five were married and four were single. Fourteen (88%) informants were from remote rural areas, seven (44%) had lived with fistula for more than six years, four (25%) were illiterate and only two (12.5%) had education beyond primary school. The age distribution of the 151 hospitalised women affected by obstetric fistula recruited in the survey ranged from 16-72 years. Characteristics of the sample are presented in Table 2.

**Table 2:** Descriptive statistics of surveyed women

Factors	Number	Frequencies (% )
**Age at Interview**		
<18	21	13.9
18-20	25	16.6
21-30	59	39.1
31-40	32	21.2
>40	14	9.3
**Total**	**151**	**100.0**
**Education level**		
Illiterate	48	31.8
Primary school	99	65.6
Secondary school & above	4	2.6
**Total**	**151**	**100.0**
**Marital status**		
Married	98	64.9
Single	24	15.9
Divorced	27	17.9
Widowed	2	1.3
**Total**	**151**	**100.0**
With whom do you live*		
Husband/partner	90	42.7
Parents	35	16.6
In-laws	11	5.2
Children	33	15.6
Relatives	35	16.6
Alone	7	3.3
**Total**	**211**	**100.0**
**Age at first delivery**		
< 18	62	41.1
18-20	40	26.5
21-30	44	29.1
> 30	1	.7
Missing data	4	2.6
**Total**	**151**	**100.0**
**Parity when fistula occurred**		
First	84	55.6
Second	18	11.9
Third	13	8.6
Fourth	14	9.3
Fifth and above	22	14.6
**Total**	**151**	**100.0**
**Lived with fistula**		
< 1 year	86	57.0
1-2 years	28	18.5
3-6 Years	17	11.3
> 6 years	20	13.2
**Total**	**151**	**100.0**

*≥ 1 responses

### Jemimah's story

The story of Jemimah (not her real name) presented below gives an example of the difficult birth experiences described by many women. It is important to note that the story is based on Jemimah's experiences and the way she retrospectively interpreted them.

Jemimah is a 20-year-old woman from Morogoro. She gave birth and her baby died four months later. Since then she has been leaking urine and faeces through her vagina. She is single, has no live child and is living in a family house together with her mother, uncle, grandmother and siblings. She did not complete primary school and had to quit her job as a house girl when she got pregnant. Because of her situation, she is currently unable to work.

Jemimah was aware of the importance of attending antenatal clinic during pregnancy and attended four times for routine check-ups as she had been advised. She had planned to deliver at the local dispensary. When labour started, however, her uncle called the traditional birth attendant (TBA) who came to assist her giving birth at home. When the sun rose the next morning, Jemimah had spent the whole night at home with the TBA without any progress of labour. The TBA realised that she could not help and advised Jemimah to seek help at the village dispensary. Her mother accompanied her there. On arrival, Jemimah remembers being asked if she was in labour and replied that, she was just feeling pain around the waist. She was surprised that she was told to start pushing without having had any kind of assessment of her condition first. Jemimah tried to follow the instructions. She started pushing, she pushed and pushed, but there was no progress. The nurses at the dispensary got worried. They did not know what to do so they called other people in the village to help: *"Some put cloths into my mouth; others tightened my legs, while others pinched me, as they were telling me I must push. In the end, I was exhausted, and foam started coming out of my mouth. There was no progress; the baby was stuck in the pelvis, when I raised my legs the baby could easily be seen. It was stuck between my legs. After many hours my legs became numb and I could not walk"*. The second day of labour passed. Jemimah was told that the doctor would come to help her, but she was not told what the problem was: *"On the third day, the doctor came to examine me in the afternoon. He told me that the baby was dead in the womb. Shortly after, I started to bleed heavily from the vagina. The doctor then gave me an injection on my thigh and we were told to go to Morogoro Hospital"*. Morogoro Hospital is about four hours by car from the village. Jemimah's mother had no money for transport, but finally managed to borrow a car that took them to Morogoro Hospital where the dead baby was removed by forceps many hours later. The following day Jemimah was shocked to notice that both stool and urine were leaking through the vagina.

### Deciding where to give birth

Almost all women in this study explained that they had planned to give birth in a health facility. However, decisions concerning where to go seemed to be taken by others and not by the woman herself. According to the quantitative survey findings (Table 3), the woman herself only decided in 7% of cases, while the husband and other in-laws, mostly mothers-in-law, made the decision in 60% of cases. In the qualitative interviews, informants explained that their husbands and mothers-in-law were inclined to prefer home delivery for a number of reasons, including convenience, custom and cost.

**Table 3:** Birthing experiences of surveyed women

	Frequency	%
**Place of delivery**		
Home	10	6.6
Traditional birth attendant (TBA)'s home	7	4.6
On the road	5	3.3
Dispensary/health Centre	12	7.9
Hospital	117	77.5
**Total**	**151**	**100**
**Who assisted during labour**		
Doctor	72	47.7
Midwife	54	35.8
TBA	12	7.9
Family members	12	7.9
Others	1	0.7
**Total**	**151**	**100**
**Who decided where to deliver**		
Husband	68	45.0
Mother in law	23	15.3
Mother	31	20.5
Father	14	9.3
Self	10	6.6
Traditional birth attendant	5	3.3
**Total**	**151**	**100**
**How did you get to the health care facility**		
By foot	28	18.5
Carried	2	1.4
Bike/public transport	65	43.0
Ox-cart/donkey/horse	1	0.7
Private car	44	29.1
Others	11	7.3
**Total**	**151**	**100**
**How many times did the sun rise from the onset of labour until they reached place of delivery**		
0	1	0.7
1	50	33.1
2	47	31.1
3	27	17.9
4	3	2.0
Missing	23	15.2
**Total**	**151**	**100**
**How many times did the sun rise from the onset of labour until the baby was out**		
1	38	25.2
2	37	24.4
3	41	27.2
4	9	5.9
5	1	0.7
Missing	25	15.5
**Total**	**151**	**100**

The women started the birthing process at home and sought skilled care in a health facility only if labour did not progress as expected. Many of the women had to accept giving birth at home, because they did not have money of their own to cover the expenses for a health facility delivery. Income poverty combined with limited decision-making power, appeared to present them with no choice but to give birth at home. Women who depended on their husband's consent to deliver in a health facility had to wait for him to return from other chores before a decision on place of birth could be reached. If he was not around, no one could make the decision. As one of the women explained:

*"I planned to deliver at the hospital but it was not possible because I did not have cash and I did not know where I will get money from. It was January, during the farming season, I did not go to the field, my husband went, I did not go, and it was then that I got these problems" *(35 year-old from Manyoni).

In many places, home birth is customary for most women. Their grandmothers and mothers had given birth at home and consider home birth to be normal and safe. Furthermore, their close kin commonly knew TBAs and had great faith in their delivery skills. As described by a woman who blamed the TBA for her birth injury:

*"My parents said I should wait as I could deliver at home...you know in the village people do deliver in homes (...) in our village, there is a TBA, She is the one who harmed us" *(35 year-old from Kisongo, Kilwa).

Another important issue influencing the choice of birthing place that came up during the interviews was the fear of humiliation from staff at the health facility. During antenatal clinic check-ups, the nurses told many women, especially the primigravida and the grand multipara, that when labour starts they should go to the district hospital to give birth. If the women returned to the same health facility for delivery despite this advice, they feared that the nurses would treat them as irresponsible and stupid.

As one of the women explained:

*"(...) when labour started, I did not go to the dispensary because I was told to go to Manyoni Hospital to give birth. Now if I would have gone back to the dispensary they could have embarrassed me" (*40 year-old from Mbugani).

Hence, there are factors emanating from the local community as well as from the health care system that tend to motivate home delivery.

### Struggling to reach a health facility

Only 74 (49%) of the women arrived at the final place of delivery within two days, and half of them (51%) arrived after two or more days. We found significant associations between early attendance at health care facilities after the onset of labour and women's educational level (*Chi-square= 20.19, p <0.05)*, and living with a husband *(Chi-square= 12.34, p <0.05). *However, we did not find significant associations between early attendance and women's age or marital status. Sixty-five women (43%) reported travelling by public transport, while 30 (20%) reported that they had walked or been carried to the health facility (Table 3). Most of the women interviewed lived in remote rural areas, and not all villages were privileged to have a dispensary nearby. Long distances to the health facilities, unavailability of public transport, and failure to meet transportation costs caused the labouring women to spend a long time on the road. One woman explained:

*"In our village there is no dispensary and there was no transport, we had to walk for three hours to get to Mvumi Mission Hospital. I had very strong labour pains and after about two hours walk, I felt like something had ruptured in the womb. I started bleeding and we had to stop and rest before I could manage to continue walking" *(28 year-old from Mvumi, Dodoma).

Another woman experienced being stuck at home because there was no transport available:

*"(...) from our village to Iseke (midway to Manyoni Hospital) is 24 kilometres, and from Iseke to Manyoni is very far, and there was no means of transport. Therefore, when labour pains started, everybody was confused because I could not walk, and there was no means of transport to take me to Manyoni Hospital. I therefore remained at home" *(35 year-old from Singida).

### Waiting at the health facility

As shown in Table 3, in spite of considerable delays on the way between home and health facility, 129 (85%) of respondents reported that they reached and gave birth in a health facility. However, women who reached the health facility still experienced considerable delays in getting adequate birth care. Many of the women interviewed experienced a delayed response from nurses and lack of support during the birth process with no progress of labour.

Women reported that they were left to push alone, without support or guidance from the nurses. Others said that in the absence of attention from the nurses, their relatives or TBAs who accompanied them to the facility were the ones that provided care and support during labour. Quantitative responses, however, indicated only a few (16%) reported being assisted by relatives or TBAs who accompanied them to the facility.

Looking back at her delivery, one woman emotionally described the problem of what amounts to negligence and arrogance on the part of the nurses. She remembered not being seriously taken care of, and was continuously told to wait while the baby was stuck in her pelvis and she was screaming in pain:

*"(...) they placed me on a labour bed, and they just sat there chatting, when you yell with pain, they say you just wait, shouting from where they were, "you are not yet ready for delivery", so I kept on waiting while being tortured with pain" *(28 year-old from Mbezi, Dar es Salaam).

Several women complained about poor referral routines. It seemed that nurses tended to delay the decision to consult doctors or to timely transfer labouring women to higher-level health facility for appropriate birth care after noticing that spontaneous delivery was difficult. As one of them said:

*"(...) in the dispensary, they did nothing; they did not check anything on me. I was left alone....I was alone throughout the night, there was no doctor... they took me to Pande hospital in the morning, by then labour pain had ceased already" *(35 year-old from Kilwa Kisongo).

Women consistently reported that in the health facility, they were left to wait for many hours or even days before the decisions to refer to the higher-level health care facility were made. This was closely linked to the division of labour between the professional groups working at the health facility. Doctors or clinical officers routinely decided upon referrals. If the doctor was not around, the patient had to wait for the referral letter. One woman narrated how she waited for almost 24 hours before a doctor (clinical officer) could see her:

*"When I got to the dispensary nurses told me to wait. At 8 pm, labour pains became intense, I started pushing but the baby could not come out, and the doctor was not around. The next day I continued pushing the whole day again. The following day in the evening is when the doctor came. He inserted his hand and started pulling the baby. Only the head came out, the entire body was left in the womb that is when the doctor referred me to Mpwapwa hospital" *(33 year-old from Mbori, Dodoma).

Another woman complained about a similar situation where she felt neglected and left alone until it was too late:

*"Labour pain started at home at around 1am. I stayed until at 3am when we found the pains were picking up, we took a taxi to dispensary. In the dispensary, I slept, spent the day, slept, and woke-up again, on the third day it was when a decision was made to transfer me to a big hospital. They said we are failing here because you have urine retention. That was when I was transferred to Tumbi hospital" *(20 year old from Mlandizi, Pwani).

### Receiving unskilled care

The experience of nurses negligence and indifference to the pains and needs of the labouring women, combined with poor monitoring and referral routines, were part of the greater picture of unskilled birth care provided in lower level health facilities. A 32 year-old woman described how nurses had forced her to push:

*"Nurses told me 'push, push' while blocking my mouth, whereas others were pressing on my abdomen. It was a very painful experience, I tried to complain, and said look, you will kill me, it is better for you to leave me and let me die. (...) at that time I felt that instead of the baby coming down, it was moving upwards, and that is when I started experiencing numbness on my leg. That was the time they stopped and called the doctor" *(32 year-old from Mang'ula, Morogoro).

In two cases, including that of Jemimah, women reported that the nurses had requested TBAs from the village to come to the health facility to assist deliveries. According to the informants, neither the TBAs nor the trained nurses systematically monitored the progress of labour, and both groups used outdated and risky strategies such as fundal pressure to help women give birth [49].

Other women experienced frequent unnecessary vaginal examinations to the extent that the vulva swelled, preventing normal progress of labour. At the same time, frequent vaginal examinations by different people presented a serious risk of infection. The nurses seemed to lack preparedness and established routines on how to handle poor progress of labour:

*"At 9 pm in the evening, they took me to Korogwe. By that time, I was in critical condition and the baby's head could be visualised, but could not come out because the vulva was swollen. Everybody was trying to insert a finger -everybody was trying to help. As you know in the village, they were asking themselves what to do. (...) by the time we reached Korogwe Hospital, I was unconscious" (*25 year-old from Mbale, Tanga).

According to the survey results, 80 (53%) of the women finally delivered by caesarean section in a referral hospital and of these 68 (85%) lost their babies. Only 20 (13.2%) of the babies delivered were alive and healthy at the time of data collection.

## Discussion

After methodological considerations, the discussion focuses on the three delays, and how these delays interact with AAAQ to produce vulnerability to obstetric complications.

### Methodological considerations

These findings are based on accounts and views of the women affected by obstetric fistula. Because health care providers were not interviewed, this could give a narrow understanding of women's experiences of birth care, especially in the health care facilities. Some women in this study had lived with fistula for many years and may not have accurately remembered the events that led to a birth that happened many years ago. There is a possibility of over-reporting their negative experiences of birth care, and that they could have assimilated the experience of others into their own. However, their openness and their immediate and consistent responses to the questions and probes during interviews left no doubt that these women recalled their birth experiences vividly.

Research has shown that women remember the birthing event and feelings surrounding labour and delivery for many years even when the process is uncomplicated [50]. While the sample used in this study only included women who had bad birth care experiences, it allowed us to document the birth care experiences of this particular group of vulnerable women who are those least to be heard. The findings cannot be generalised, as the selection of participants in the quantitative study was not random. Nevertheless, this study included the women who were affected by obstetric fistula from different regions of Tanzania; the experiences upon which the findings were documented have relevance in other contexts that have similar social economic characteristics as Tanzania.

### Delays occurring at home

Lack of women's decision-making power on where to seek obstetric care, the distance to health facilities and lack of money contributed to the first and second delays (seeking adequate care and identifying a health care facility to go to). In Tanzania, like in most societies in sub-Saharan Africa where the dominant social structure is patriarchal, the paternal family tends to dominate the decision-making processes [51,52], and birth care is no exception. The paternal family is expected to cover the costs, and thus has the final say [53]. In this study, husbands and mothers-in-law had a key role in deciding when and where obstetric care should be sought. Many women were not involved at all in arriving at the decision to seek care, whether from the TBA or at health facilities. Similar findings were found in studies carried out elsewhere [54,55]. Although the great majority of women (85%) ended up in health care facilities and only a few (15%) gave birth at home, this was often after considerable delay and poor progress of labour. Previous research established that husbands' permission was needed before TBAs could refer a woman to the health facility [56,57]. Women may have to wait for permission to seek birth care from their husbands even when the health facility is nearby, causing major delays at home [9].

Women's inability to make decisions as documented in this study may be related to their lack of economic empowerment, as noted in other studies [52,58]. In addition, women's decision-making power could be affected by their illiteracy. This may have a direct implication to the women's involvement in unskilled and less paid jobs or subsistence farming. These women therefore rarely have cash available at all times for emergencies unless given money by their husbands. It has been observed that husbands, as heads of households, commonly control household income [51].

### Delays in identifying and reaching a health facility

The unavailability of money, distance to health facility or lack of transport, and the presence of TBAs were all identified barriers that prevented women from seeking adequate birth care, in spite the women's intent to give birth in a health facility.

Distance to the health facility was the most important impediment, even in situations where the decision to seek adequate care had been made. Although this study did not establish the actual distance these women walked to the nearest health facilities, the study findings support previous research findings [24,56,57,59-63]. The cost of accessing care is a critical determinant of whether or not professional care is sought. The probability of home birth increases: if an adequate health facility is far away from where women live, if infrastructure for transport is lacking, and if the community is poor. Even though the National Health Policy of Tanzania aims to provide a dispensary to cater for 5000 people and oversee all village health services [13], not all villages have been provided with this basic health facility.

### Delays receiving adequate care in health facilities

From a health systems point of view, the most serious delays were encountered after arrival in a health facility. This study illuminates the problem of unskilled birth care offered to women in health facilities. These findings are corroborated by studies done elsewhere [54,64-66]. Inadequate skilled attendance at delivery is one of the key indicators that reflect poor progress towards the MDG 5 of improving maternal health. In Tanzania births attended by skilled attendants are still low [5] and availability of skilled attendants without accessibility of emergency obstetric services also plays a role in negative maternal outcomes [17]. Poor access to emergency obstetric care (EmOC) services and poor quality of care offered in lower level health facilities have probably contributed to women's low expectations to maternal health services and undermined the trust in the health care system. Lack of trust negatively affects health care utilisation and therefore is a major threat to the achievement of MDG 5.

A woman who, after struggling to reach a health facility, finds that she receives the same services as those available at home, might not choose to return to the health facility the next time. The practice of inviting TBAs to offer assistance inside the health facility may not be common, but when there is a severe health worker shortage, it may simply be linked to the need for more hands. It may also indicate the need to share responsibility for the birth outcome. This practice however, blurs the boundary between professional and lay care, and transfers authority from the professional health worker to the TBA, thus giving legitimacy to the work and the role of TBAs, both within and outside the health care facility. Nearly all women interviewed in the qualitative study reported that they sought advice or care from TBAs at some point. However, based on the findings on the role of TBAs in bringing down maternal mortality, the government of Tanzania no longer recognises TBAs as partners in maternal health and has discontinued training and collaboration with them [67]. Nevertheless, other studies have proposed that the training of TBAs could be beneficial as a strategy to reduce the first delay [57,68].

The women interviewed in this study perceived provision of substandard care in the health facility as arising from negligence on the part of health care providers. Many women reported missing adequate attention and support from nurses, as they were left alone during the birthing process. About 16% of the women in the survey reported that they received birth care from their relatives while in the health facilities and health care providers were not responsive even when called to assist. Even so, the great majority of women (85%) ended up having their delivery in a health care facility, but more than half (51%) had resorted to these facilities only after two days or more of labouring at home. This could indicate lack of trust in the health care system or it could also indicate a birthing culture in which health facilities are resorted to, only during complications.

Another study done in Tanzania addressing the issue of trust indicated that mothers often bypass a nearby health facility and go to one that is far away if it is perceived to give better quality of care [19]. The lack of support from health care providers during labour may lead to anxiety and subsequent dissatisfaction with the birth process [69]. As suggested by Field [70], the actions of health care providers are often more important for patient satisfaction than the physical environment.

The experience of negligence and poor quality of care contributes not only to the third delay (receiving inadequate care) but is also a persuading factor for the first and second delays (seeking adequate care and identifying a health care facility to go to). Women's experiences of birth care at health facilities push them towards home deliveries and reinforce their reliance on TBAs. Studies have demonstrated that provision of poor quality of care by nurses prohibits women from seeking skilled care [61,63-65].

It is generally assumed that trained health care providers (nurses, clinical officers, and doctors) provide skilled delivery assistance. Women in this study experienced poor quality of care even while at higher-level health facilities where well-trained health care providers are supposed to be available. Although this study did not interview health care providers, the accounts of the women point to a major health systems failure that needs urgent attention.

The poor quality of care experienced by the women in this study could be linked to lack of commitment on the part of the health workers and/or a disabling working environment. Inadequacy of equipment and materials, coupled with the long working hours, absence of opportunities and lack of support from either their supervisors or fellow staff may disable health care providers to offer the services expected, and work to leave them powerless when complications occur.

Commonly, due to the shortage of skilled staff at lower level health facilities, only one health care provider was present at the time to provide care to all patients and clients in all sections of the health facility. Consequently, in case of difficulties, the health care providers have no one to consult. Working in isolation tends to negatively affect motivation [69]. As observed by Hussein and others, skilled attendants require an enabling environment with availability of resources including staff, drugs, equipment, supplies, motivation and solid health systems to be able to provide better services [71].

### Delays in referral

Using a partograph to monitor women's progress of labour facilitates timely decision-making and referral, which are key factors in preventing obstructed labour and thereby preventing obstetric fistula [72]. Some women in this study were transferred half way through labour from one level of health care to another. Although there are often limited alternatives or choices within health care system when complications arise, early referral could help, but this is only possible if there is close monitoring and support. Carelessness and lack of capacity to foresee difficult labour in the first place may contribute to delays in the decision to refer.

No less important are rigid routines, where only the doctor or clinical officer, (who may be absent) is authorized to endorse referrals. In addition, most rural health facilities have no communication mechanism that can facilitate efficient referral, once the decision has been made. Ambulance services are not available in these first-level health facilities, and in areas where transport is available, it is the responsibility of the family to pay. Others [53,64] have reported this phenomenon.

The findings of this study, including Jemimah's birth narratives, illustrate how delays occur and are experienced in each phase of the birthing process, and how these delays can be detrimental to the outcome of labour for both the mother and the child. This study also reveals a series of weaknesses in the health care system associated with obstetric competence, infrastructure and health worker-patient relationships.

## Conclusion

In this study of women's birth care experiences, delays in receiving adequate care after arrival at health facilities emerged as the most central finding in women's accounts. In line with Thaddeus and Maine [9], we argue that the three delays are closely connected and that delays after arrival at health facilities fundamentally undermine trust and causes delays in the decision to seek professional care in the future. The findings related to delays at home and during transportation provide a fuller picture of women's birthing experience, and illustrate the relative importance of the different delays as reflected in women's accounts.

This is of particular importance in view of the current policy to increase institutional deliveries in Tanzania as a means to bring down maternal mortality and morbidity (in the process to reach MDG 5), and in a context where the MoHSW in collaboration with NGOs work to motivate women to deliver in hospital. The delays prior to arrival in health facility cannot be expected to change until the quality of care offered in health facilities has improved.

The acceptability and the quality of the existing health services must be strengthened and professional ethics systematically incorporated into the training of health care providers. Existing resources, guidelines and tools to monitor progress of labour such as partographs should be reinforced. In addition, accountability must be ensured through better reporting and follow-up routines.

Women's access to health care is a human rights agenda [42]. To address the problem of delays in receiving adequate birth care, the government needs to secure EmOC that is available, accessible, acceptable and of adequate quality also for women living in rural Tanzania.

## Competing interests

The authors declare that they have no competing interests.

## Authors' contributions

LTM developed the concept of the study and the design, also organised and collected qualitative and supervised quantitative data collection. LTM, KMM and TWK did the data analysis and interpretation. LTM drafted the article, which was then critically reviewed and revised by TWK, KMM, BEO and AM. All authors were involved in the discussions during the planning and follow-up phases of the study and the development of the quantitative questionnaire at the (GeSoMo) - NUFU workshops. All authors read and approved the final manuscript.

## Pre-publication history

The pre-publication history for this paper can be accessed here:

http://www.biomedcentral.com/1471-2393/11/75/prepub

## Supplementary Material

Additional file 1 **Questionnaire**. This is a four-section questionnaire, in PDF format that was used to collect data on challenges and experiences of women affected by obstetric fistula in Tanzania. Click here for file
