# Supplementary material for: Waiting for attention and care: birthing accounts of women in rural Tanzania who developed obstetric fistula as an outcome of labour
Source: BMC Pregnancy Childbirth. 2011 Oct 21;11:75. doi: 10.1186/1471-2393-11-75 (PMC3221614; doi:10.1186/1471-2393-11-75)
Supplement: Additional file 1 — Questionnaire. This is a four-section questionnaire, in PDF format that was used to collect data on challenges and experiences of women affected by obstetric fistula in Tanzania. [file 1471-2393-11-75-S1.PDF]

**Addis Ababa University, Ahfad University for Women, Bergen University College, Muhimbili University of Health and Allied Sciences, University of Bergen and University of Dar-Es-Salaam,**
**NUFU Project: Gender, Generation and Social Mobilization: Challenges of Reproductive Health and Rights among Vulnerable Groups in Ethiopia, Sudan and Tanzania**

---

**Muhimbili University of Health and Allied Sciences**
**School of Nursing**
**Challenges & Experiences of Women affected by Obstetric Fistula in Tanzania (Questionnaire Survey)**

## A. Socio-demographic characteristics

1. How old are you? ___________

2. Educational level
   1. Illiterate
   2. Read and write
   3. Primary (1-7)
   4. Secondary O level (Form I-IV)
   5. Secondary A level (Form V-VI)
   6. Above secondary education, specify………..

3. What is your marital status?
   1. Married
   2. Cohabiting
   3. Widowed
   4. Separated but not divorced
   5. Divorced
   6. Never Married
   7. Other, please specify……

4. With whom do you live? (More than one option can be ticked off)
   1. Your husband ☐
   2. Live-in partner ☐
   3. Your parents ☐
   4. Your in-laws ☐
   5. Your children ☐
   6. Your Brothers/ sisters ☐
   7. Other relatives ☐
   8. Non family members ☐
   9. live alone ☐
   10. Other, please specify………..

5. How old were you when you first got married? ______________

6. How old were you when you delivered for first time? _______

7. In which pregnancy did the fistula occur?
   1. First pregnancy
   2. Second pregnancy
   3. Third pregnancy
   4. Fourth pregnancy
   5. Other, Please specify...............

8. For how long did you stay with obstetric fistula before treatment?
   1. Months (           )
   2. Years   (           )

## B. The health structure / care

(Questions in this part apply to the pregnancy & delivery related to the fistula)

9. Where did you deliver your child?
   1. Home
   2. In a TBA's house / clinic
   3. Hospital
   4. Health centre / Dispensary / health post
   5. On the road
   6. Other, please specify............

10. Who assisted you during labour?
    1. Doctor
    2. Midwife
    3. Traditional birth attendant
    4. Family members
    5. Others please specify............

11. Who made the decision as to where to go to seek help during the last delivery?
    1. Husband
    2. Mother in law
    3. Mother
    4. Father
    5. Yourself
    6. TBA
    7. Other, pleas specify.........

12. How did you get to the health care facility (if applicable)?
    1. by foot
    2. Bike / Bus / minibus / Taxi (public transportation)
    3. Ox-cart / Donkey / horse
    4. Private car
    5. Carried
    6. Other, please specify .........

13. How many times did the sun rise from the onset of labour till you arrived at the final place of delivery (referral time, if applicable) ___________________

14. How many times did the sun rise from the onset of labour till the baby was out? ________

15. How was the baby delivered?
1. Without operative instruments
2. Vacuum / forceps
3. Caesarean section
4. Baby removed little by little
5. Spontaneous
6. Don’t remember
7. Other ________________

16. What was the condition of the child after delivery?
1. Alive and healthy
2. Alive, but in bad condition
3. Alive, but died later
4. Dead
5. Other, please specify ________________

## C. Nutritional status:

17. In addition to yourself how many people live in the house where you currently stay?
1. 0
2. 1-3
3. 4-6
4. 7-10
5. > 10

18. When you were a child how many meals did you normally ate per day?
1. One
2. Two
3. Three
4. More than 3

19. During pregnancy how many meals did you normally eat per day?
1. One
2. Two
3. Three
4. More than 3

20. How many meals do you normally eat per day now?
1. One
2. Two
3. Three
4. More than 3

21. How do you eat your meals?
1. Alone

2. with others
3. It varies from day to day
4. Other, please specify ______________

22. What type of food do you normally eat?
1. ___________________________
2. ___________________________
3. ___________________________
4. __________________________

23. Have you been restricted from any type of food/drinks after developing the fistula?
1. Yes
2. No

24. If yes, what types of foods/drinks and why?
1. _____________________________
2. _____________________________

25. Who gives you food / money for food?
1. No one
2. Husband /partner
3. Family / relatives
4. Neighbours /friends
5. Others please specify ............

26. How do you consider your stature (Physique, size) comparing to your family members?
1. Shortest
2. Tallest
3. Normal
4. Other (specify .........)

27. Among your siblings, in which order were you born?
1. First born
2. Second born
3. Third born
4. If other, (specify order...............)

28. What is the age difference between you and your immediate siblings?
1. Older sibling (numbers of years).........
2. Younger sibling (number of years).........
3. Don’t know

## D. Reintegration

29. Did you go back home after surgery?
1. Yes
2. No

(If yes ask question 30 if not go to question 31)

30. How were you received when you returned back after the first surgery (if applicable)?
    1. Happily / friendly
    2. With reservations
    3. Unfriendly
    4. I do not know
    5. Other, please specify ...............

31. Where did you go to stay?
    1. With friends
    2. Relatives
    3. Neighbours
    4. Other Specify

32. With whom are you planning to stay after the surgery has been completed?
    1. Your husband
    2. Your live-in partner
    3. Your own parents
    4. Your parents in law
    5. Relatives
    6. Others please specify...............

33. Who do you think will accept you?
    1. Your husband ☐
    2. Your live-in partner ☐
    3. Your own parents ☐
    4. Your parents in law ☐
    5. Children ☐
    6. Other relatives ☐
    7. Neighbours /friends ☐
    8. Community / church ☐

34. In the future, do you hope to give birth again?
    1. Yes
    2. No
    3. Don’t know
    4. Not possible

35. In the future, would you like to marry again (if applicable)
    1. Yes
    2. No
    3. Don’t know

36. How do you see yourself 5 or 10 years from now?
    1. Living with husband and children
    2. Living with relatives
    3. Living alone

4. Other, please specify…………

37. How do you see yourself make a living?
    1. Having own job / income
    2. Help from family/relatives
    3. Help from NGO or other organization
    4. Other, please specify…………………

**Ht (cm)…………… Wt (kg) ……… BMI…………**

Thank you so much for your participation!
